# Clinical complications during treatment with a modified Herbst appliance in combination with a lingual appliance

**DOI:** 10.1186/s13005-015-0088-3

**Published:** 2015-09-09

**Authors:** Dirk Wiechmann, Julius Vu, Rainer Schwestka-Polly, Hans-Joachim Helms, Michael Knösel

**Affiliations:** Orthodontic Practice, Lindenstrasse 44, Bad Essen, 49152 Germany; Department of Orthodontics, Hannover Medical School (MHH), Hannover, 30625 Germany; Department of Medical Statistics, University Medical Center Göttingen (UMG), Göttingen, 37099 Germany; Department of Orthodontics, University Medical Center Göttingen (UMG), Göttingen, 37099 Germany

**Keywords:** Herbst appliance, Complication, Fracture, WIN appliance, Angle Class II, Lingual orthodontic treatment, Survival rate, Kaplan-Meier

## Abstract

**Objective:**

To assess the types and frequencies of clinical complications experienced when using a modified lingual Herbst appliance and to compare these with those associated with conventional Herbst appliances reported in the literature.

**Methods:**

Treatment records for 35 consecutive subjects treated during the observation period from October 2013 to August 2014 who received a combination of a lingual appliance and a modified Herbst appliance (WIN, DW LingualSystems) were assessed for complications linked to Herbst treatment phase. Complications were analyzed descriptively, and complication-free intervals were calculated using Kaplan-Meier plots. To enable a comparison with data reported in the literature, the cumulative treatment time for all subjects was divided by the total number of complications.

**Results:**

71.4 % of Herbst treatments were free from complications (*n* = 25). Complications were seen on 13 occasions (8 instances of Herbst attachment loosening, 5 L-Pin fractures). Most of these complications could be fixed chair side utilizing simple clinical measures. Considering all complications as identical statistical events, the percentage of treatments free from complications would be 88 % for 100 days, 70 % for 200 days and 56.8 % for 300 days. For severe complications, the averaged complication-free treatment interval was found to be 27.8 months.

**Conclusion:**

In terms of clinical sturdiness, and taking into consideration the step-wise mode of activation used here as well as the differences in the design of the various Herbst appliances, the WIN-Herbst appliance was found to be superior to comparable vestibular Herbst appliances, as well as the banded Herbst appliance belonging to the preceding generation of customized lingual systems. Success in treatment of non-compliant Angle Class II correction is considered to have better predictability using the modified anchorage strategy of the WIN-Herbst appliance.

## Introduction

Angle Class II category dentofacial deviations are one of the most prevalent malocclusions, with a proportion of 20–32 % [1, 2].

Angle Class II malocclusions are often accompanied by a sagittal retrusion of the mandible and are therefore commonly treated by positioning the mandible forwards, to achieve dental and skeletal changes [3]. Depending on the extent of the malocclusion, subject’s age, and remaining dentofacial growth potential, a variety of both removable and fixed appliances are available for Angle Class II correction. The latter are often used in combination with extraction therapy, Class II elastics, or flexible or rigid fixed functional appliances [4, 5]. In cases of severe Angle Class II malocclusions following completion of dentofacial growth, orthognathic surgery of the mandible is often considered to be a viable method of therapy. Otherwise, the Herbst appliance is considered to be an integral part of the current orthodontic therapeutic spectrum [3, 4, 6–11].

A typical weak spot of many fixed functional appliances for class II-correction is their type of anchorage or attachment: Archwires and brackets at the insertion site of functional appliances are most likely to be affected by fractures or failure in systems using archwires as a support. These are frequent sources of complications and often necessitate additional, time-consuming appointments for repair. Therefore, vestibular Herbst telescopes are often not supported by archwires, but instead use splints or cast bands as an anchorage. The proportion of complications or fractures with this type of appliance is reported to vary between 58 % and 88 % during treatment or during observation, respectively [12–16].

A clear tendency of an increasing proportion of adult orthodontic patients has been seen, resulting in an increased use of lingual orthodontic appliances. However, as there has also been an increased use of lingual appliances in more complex treatments in adolscents [17], combination of lingual appliances with a Herbst type functional appliance are a common element of the contemporary orthodontic spectrum of therapy [18–20]. Similarly to vestibular Herbst derivatives, the fixation of Herbst telescopes in common lingual appliances (Incognito, 3 M Unitek, TOP-Service für Lingualtechnik, Bad Essen, Germany) is achieved by bands on the lower canines and upper first molars, which are simultaneously provided with a lingual bracket [21].

**Fig. 1:**
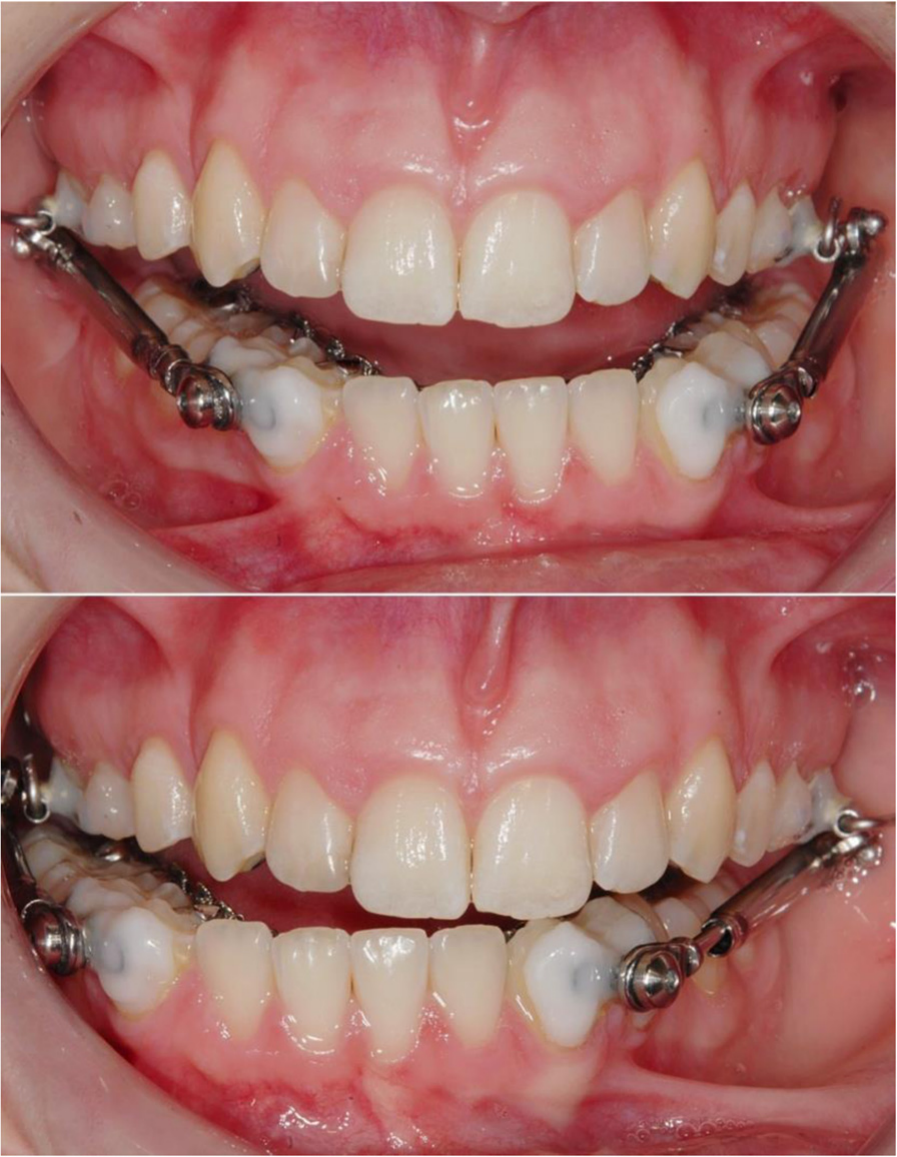
Modified Herbst anchorage using vestibular-attached shells provides increased lateral degree of freedom of the Herbst telescopes

The combination of a lingual appliance with a newly designed Herbst appliance WIN (DW Lingual Systems, Bad Essen) differs from previous lingual Herbst appliances in that the attachments are attached independently by composite adhesives to the buccal sides of the lower canines and first premolars, and upper second premolars and first molars, without being supported by bands as part of the actual lingual multi-bracket appliance (Fig. 1). Apart from an improved degree of freedom of the Herbst-telescope guided movement of the mandible, a decreased proportion of fractures or complications can be achieved with the appliance.

The aim of this study was to assess types and frequencies of clinical complications experienced with a modified lingual Herbst appliance (WIN, DW LingualSystems), and to compare these findings with those reported in the literature for existing types of Herbst appliances.

## Subjects and method

**Fig. 2:**
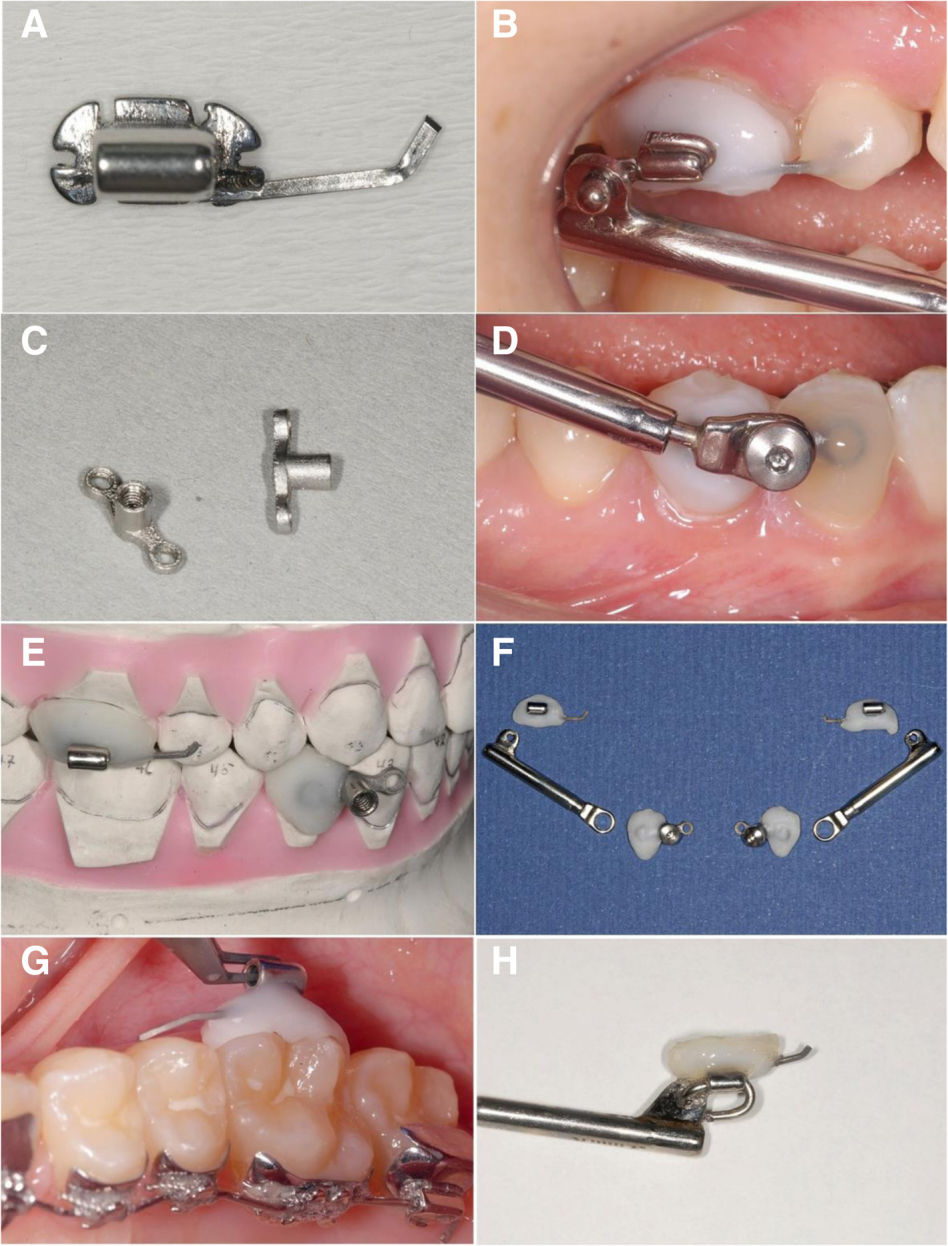
Components of the WIN Herbst appliance. Potential complications arise as a result of a loosening of the adhesive attachment shells at the upper first molars and second premolars (**a**, **b**), or at the lower canines and first premolars (**c**, **d**), potential fractures of the L-pins (**b**), or a defect in a telescope. Attachments and telescopes are individualized in the dental laboratory (**e**, **f**) and bonded directly (**g**). Loose attachments (**h**) can be re-bonded without additional dental laboratory work (restoration)

**Fig. 3:**
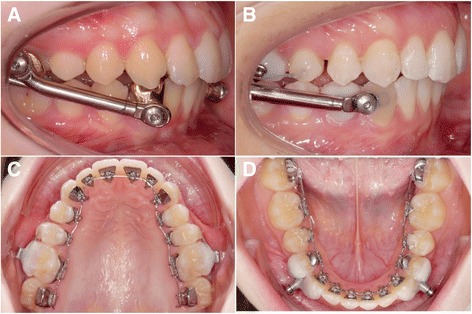
Different band-supported Herbst telescopes with a lingual multi-bracket appliance (**a**, Incognito, 3 M Unitek) compared with the anchorage concept of the investigated WIN Herbst appliance (**b**, **c**, **d**; WIN, DW Lingual Systems), which is neither directly nor indirectly linked to the archwire by any component of the appliance. Archwire fractures are a common source of complications with labial archwire-supported appliances

This retrospective study of the robustness of the modified Herbst appliance was approved by the ethics committee of the Hannover Medical School (MHH), Germany (#1220-2011). The files of all patients currently in active treatment or having undergone complete treatment using the WIN Herbst appliance with a corresponding lingual appliance (Figs. 2 and 3) in one orthodontic practice (Bad Essen, Germany) were included and screened with a caesura made on September 9, 2014. That is, the single inclusion criterion was treatment with the WIN Herbst appliance. There were no exclusion criteria other than absence of active or completed WIN Herbst treatment. The initial Angle Class II malocclusion extended to at least 3/4 cusps of distal occlusion in all subjects. Activation of the Herbst appliance was step-wise, with a final over-correction of the sagittal discrepancy.

The observation period was 10.5 months, starting on October 23rd, 2013. Based on documentation, the types and frequencies of complications associated with the incorporated Herbst appliance for a total of *n* = 35 subjects (mean age 16.9 years; female/male ratio 23 [65,71 %]: 12 [34,29 %] subjects) were assessed (Table 1).

**Table 1:** No significant differences in subjects’ ages was detected between male and female subjects (unpaired *t*-test, *p* = 0.24), nor any significant difference in Herbst treatment time between male and female subjects (unpaired *t*-test, *p* = 0.25)

	Males	Females	All Groups
	(*n* = 12)	(*n* = 23)	(*n* = 35)
	Mean (SD), [Min/Max/Median]	Mean (SD), [Min/Max/Median]	Mean (SD), [Min/Max/Median]
Subject’s age at start of MB-treatment/years	15.57 (7.63)	17.59 (7.63)	16.90 (6.28)
	[13.18/18.34/15.45]	[13.86/51.52/16.07]	[13.18/51.52/15.78]
Duration of Herbst treatment stage/months (# days/30)^a^	7.03 (1.89)	5.96 (3.57)	6.33 (3.11)
	[3.30/9.97/6.97]	[1.63/10.7/6.33]	[1.63/10.70/6.77]

^**a**^Until end of treatment or end of observation period

### Literature screening strategy

In order to retrieve relevant data related to the subject of complications following Herbst appliance treatment, an unrestricted electronic search of Pubmed was performed in December 2014. In an attempt to compensate for a holistic, systematic review of the literature using all available databases, the Pubmed search query [(Herbst) AND orthod* AND (fract* OR compli* OR fail*)] used here was provided with robust truncations, as suggested earlier by Stamm and Hohoff [22]. Title and abstract screening was performed in order to eliminate those papers not relevant to the subject of fracture rates following Herbst appliance treatment. Of a total of 39 publications, seven were identified as being relevant for a potential comparison with the findings of this study. In addition, electronic search was followed by a manual search up of the list of references in those manuscripts identified as being relevant to the subject of Herbst appliance failures.

### Statistical analysis

Complications and fractures recorded during the observation period were analyzed descriptively. Time intervals expected to be free from complications were calculated for 100, 200, and 300 days using Kaplan-Meier plots. At the end of the observation period, all subjects who showed no occurrence of an event during Herbst treatment were also censored, as information was only available for the time period between commencement of Herbst treatment and the end of the observation period.

## Results

A qualitative differentiation was made during the analysis of complication events assessed during the observation period. Complications were classified as”mild” if an easy, uncomplicated reconstitution such as the re-attachment of ready-made components or removal of sources of irritation (e.g., re-attaching of L-Pins, in analogy to a tightening of screws of comparable Angle Class II fixed functional appliances; Fig. 2), could be achieved in the same session, but without the need for new production and incorporation of parts. Complications were classified as “severe” if a loosening or fracture of individualized components (attachments or archwire) occured. Table 2 gives a list of potential complications and events which did indeed occur during the observation period.

**Table 2:** List of potential and actual complications during the observation period

Type of complication	Number of actual complications x	Frequencies of x complications, n (%)
Complications associated with mandibular canine/premolar attachments		
TOTAL loose attachment	1	2 (5.71 %)
	0	33 (94.29 %)
LEFT TOTAL loose attachment	1	1 (2.86 %)
	0	34 (97.14 %)
RIGHT TOTAL loose attachment	1	1 (2.86 %)
	0	34 (97.14 %)
TOTAL other complications	0	35 (100 %)
Complications associated with maxillary first molar (second premolar) attachments		
TOTAL loose attachment	2	1 (2.86 %)
	1	4 (11.43 %)
	0	30 (85.71 %)
LEFT TOTAL loose attachment	1	2 (5.71 %)
	0	33 (94.29 %)
RIGHT TOTAL loose attachment	1	4 (11.43 %)
	0	31 (88.57 %)
TOTAL other complications	0	35 (100 %)
Complications associated with telescopes		
TOTAL disconnected	0	35 (100 %)
LEFT TOTAL disconnected	0	35 (100 %)
RIGHT TOTAL disconnected	0	35 (100 %)
OTHERS: TOTAL others	0	35 (100 %)
L-Pin fractures		
TOTAL	1	5 (14.28 %)
	0	30 (85.72 %)
LEFT	1	3 (8.57 %)
	0	32 (91.43 %)
RIGHT	1	2 (5.71 %)
	0	33 (94.29 %)
Archwire fractures during Herbst treatment		
TOTAL	0	35 (100 %)
LEFT TOTAL	0	35 (100 %)
RIGHT TOTAL	0	35 (100 %)
Total Complications		
TOTAL Complications	2	3 (8.57 %)
	1	7 (20 %)
	0	25 (71.43 %)
LEFT TOTAL Complications	1	6 (17.14 %)
	0	29 (82.86 %)
RIGHT TOTAL Complications	2	1 (2.86 %)
	1	5 (14.29 %)
	0	29 (82.86 %)
Patients with complication	≥1 complication	10 (28.57 %)
	no complication/censored	25 (71.43 %)

During the observation period, 25 cases (71.4 %) were free from complications.

A total of 13 complications were documented in 10 subjects (28.6 %): 5 complications were fractures of the L-Pin at the upper molars which could be easily corrected, and 8 complications were loose Herbst attachments (composite shells) requiring re-attachment, of which one subject also had a fracture of an L-Pin seen at another appointment.

No archwire fractures, Herbst telescope failures, or loosening of screws were seen during the observation period.

**Fig. 4:**
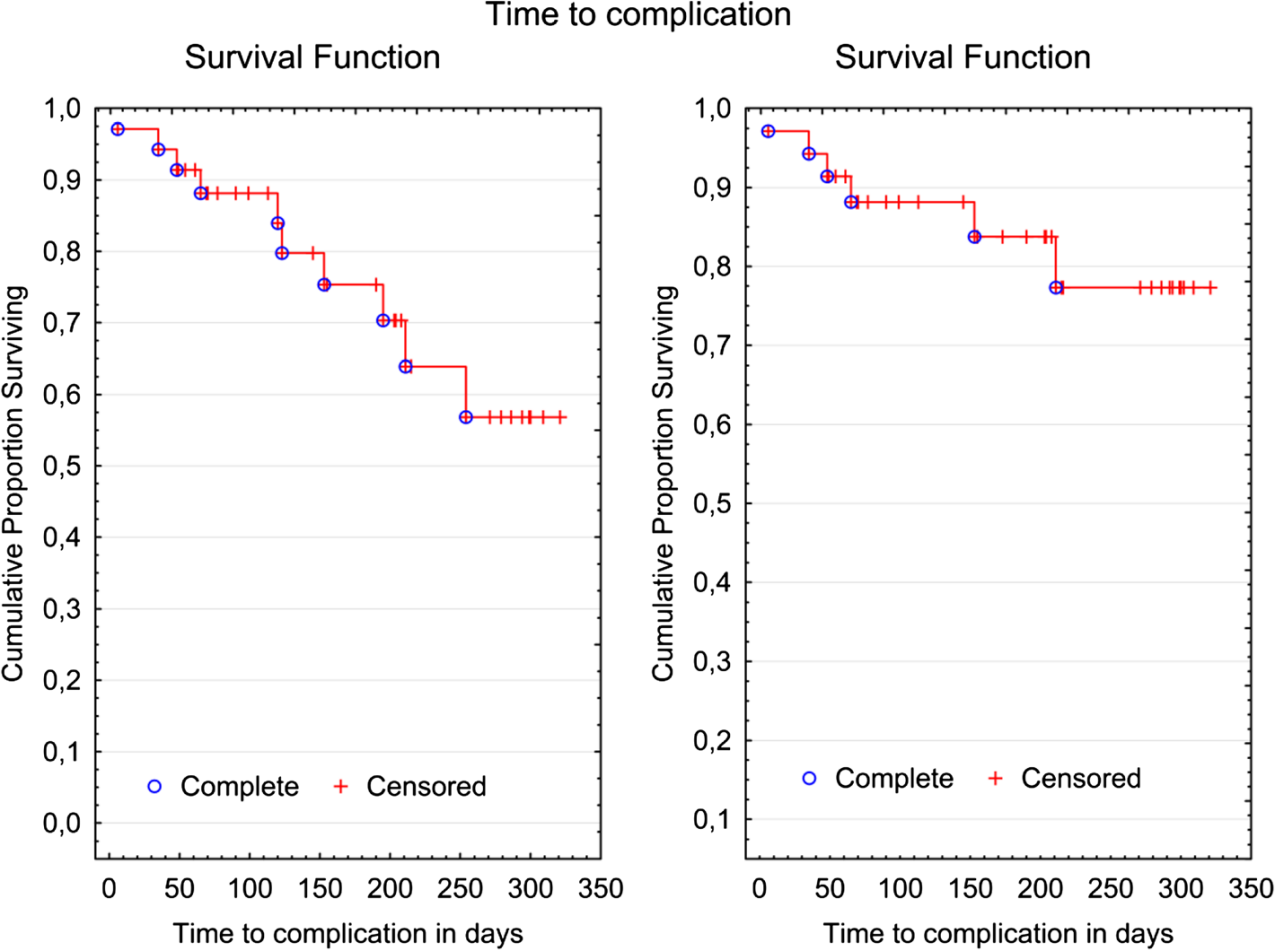
Kaplan-Meier plots: Left, Considering both minor and severe complications as equivalent statistical events, the 100 day survival rate was 88 %, the 200 day survival rate 70 %, and the 300 days survival rate 56.8 %. The median time to complication after Herbst insertion was not reached. Right, The survival rate for severe complications requiring longer appointments for repair (fractures of individualized Herbst attachments (shells), not considering L-Pin fractures; total: 6 events) was 88.2 % for 100 days, 83.7 % for 200 days and 77.3 % for 300 days

**Fig. 5:**
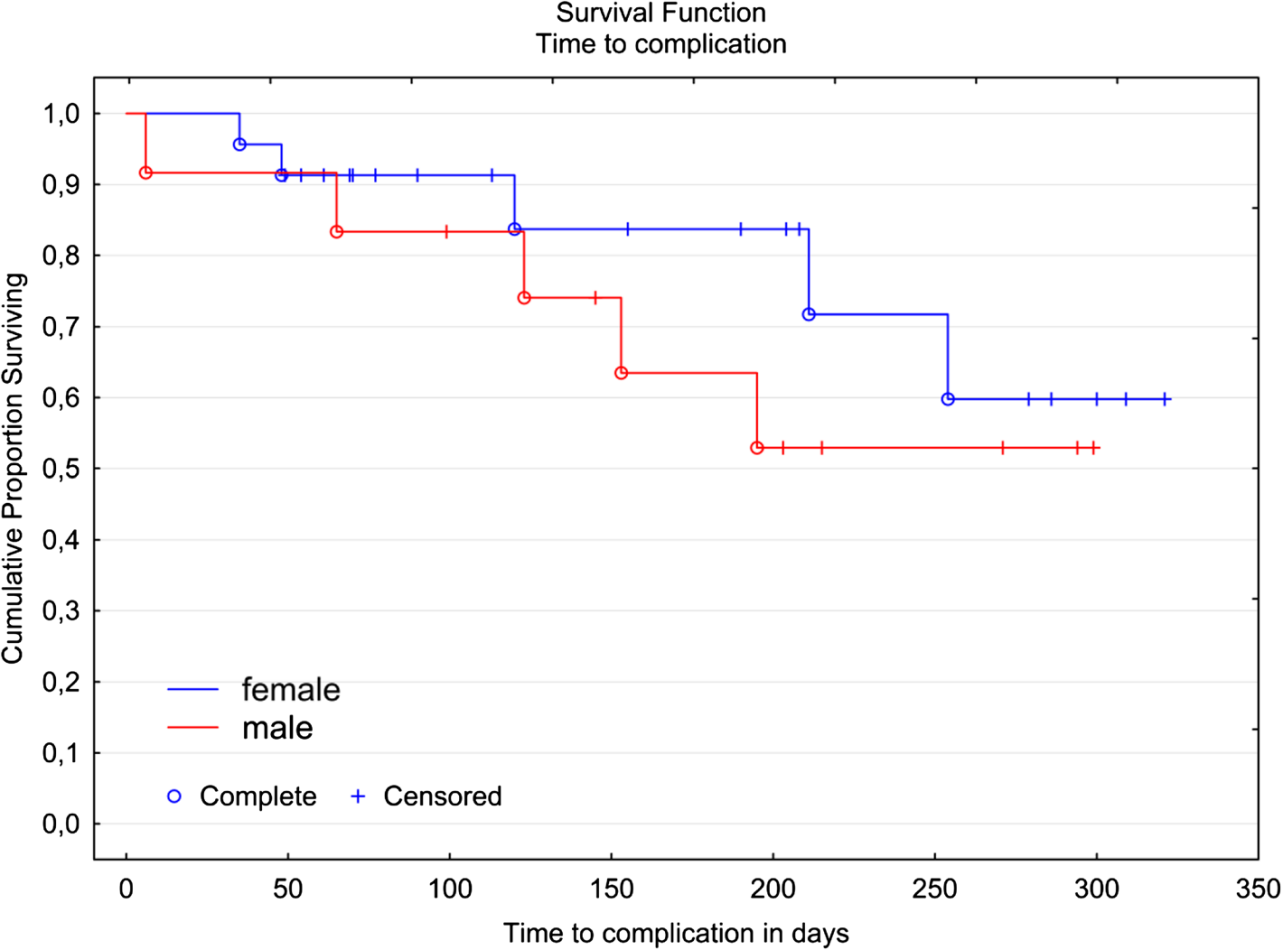
Kaplan-Meier plot: Time to any complication (including both minor and severe complications as equivalent statistical events) following Herbst incorporation, by gender (male/female). The log-rank test analyzed potential differences between the survival times for the two genders. No statistical significant differences were found in the data (*p* = 0.4). The median time to complication was reached neither for males nor females within the observational time frame. The 200 day survival rate was 53 % for males and 83.7 % for females, and 53 % or 59.8 % for 300 days, respectively

Due to the small number of total complications, the first Kaplan-Meier analysis did not differentiate between mild and severe complications, but factored all complications (those that were easy and those more laborious to correct) as identical statistical events; i.e., the fracture of an L-pin that was simply to replace was classified in the same way as a failure requiring removal, reproduction, and re-attachment of components (Fig. 4, left). No statistically significant gender-specific differences in terms of the proportion of complications (log rank test, p = 0.4) could be detected (Fig. 5). An additional Kaplan-Meier analysis was performed, providing separate depictions of the survival rate of custom-made Herbst attachments without the failure of ready-made L-pins, and the calculated complication rate (Fig. 4, right).

## Discussion

The observation time frame of this study was 10.5 months, with a mean Herbst treatment period of 6.3 months. The recommended duration of a Herbst treatment has been stated to be 10–12 months [6, 11, 23]. Therefore, proportions of treatments free of complications or survival rates were calculated for 100, 200 and 300 days using Kaplan-Meier plots (Figs. 4 and 5). Accordingly, following a treatment time elapse of 10 months, clearly more than half of cases (56.8 %) can be expected to be free from complications.

There was a considerable difference in terms of the time required to carry out clinical repairs, depending on the type of complication. Replacement of a ready-made L-pin (5 subjects) is easily accomplished and comparable to changing an elastic powerchain in patients treated with multi-bracket appliances. Re-attachment of a defective or loosened Herbst attachment (6 subjects) is more laborious and clinically can be compared to re-attaching a bracket. In every case of a complication seen in this study, it was possible to complete repairs during the same appointment, as no components needed to be manufactured in the dental laboratory.

The proportion of Herbst appliance complications has been reported to be up to 88 % during treatment time or the observation period [16]: A recent study of the complication rate for two differently attached Herbst appliances reported an incidence of complications of 85.3 % during treatment with a crown-supported appliance, and 88.0 % in subjects with an appliance that was crown-supported in the maxilla, but splint-supported in the mandible [16]; Silva et al. calculated a mean complication rate of 2.5 events per patient, with no statistically significant difference between the two appliances [16]. However, their calculated complication rate also included smaller complications not resulting in an interruption of a forward guidance of the mandible, such as gingiva irritations provoked by the Herbst appliance.

Similar percentages of complications were also reported by several other investigations regarding the robustness of Herbst appliances: Moro et al. reported a complication-free proportion of 33 % of subjects treated with a cantilever-bite-jumper Herbst appliance, and 14 % of Herbst appliances supported by a mandibular splint [13]. Latkauskiene et al. reported a total of 46 Herbst appliance fractures for a sample of 175 subjects (26.3 %) undergoing fixed functional pre-treatment prior to initiation of multi-bracket treatment in some cases [24]. Hägg et al. reported a proportion of 14 % of complication free instances for the cast Herbst appliance and 21 % for the band-supported Herbst [12]. Accordingly, the mean complication rate per case or individual treatment was reported, respectively, to be 1.1 or 2.5 by Moro et al., and 3.7 or 2.9 by Hägg et al. [12, 13].

However, it may be considered quite useful to differentiate between simple and severe complications when evaluating the proneness of a functional appliance to defects in relation to a mandible guidance that is free of interruptions and, in this context, to disregard complications such as gingiva irritations [25]. While the first group of complications does not immediately impede the functionality of the appliance, such as the loosening of a telescope screw, severe complications or appliance fractures (such as a failure of an attachment shell or archwire failure in archwire-supported systems) temporarily disrupt mandibular guidance, and prolong total treatment duration. Therefore, consideration of treatment intervals without severe complications is useful, in order to characterize the robustness of a fixed, functional appliance. This information is often not directly manifest from the literature [12–14, 16], but can be calculated by multiplying numbers of study patients by mean treatment time and then dividing this by the numbers of severe complications that occurred. Though this kind of calculation probably averages repeated events over the whole sample, which may not necessarily be related to the appliance itself (such as with repeated fractures in single patients, as was seen in this study, but also others - see Hägg et al., who reported on one subject who was affected six times by a fracture) [12]. However, bearing in mind this limitation, such a calculation gives a frame of reference for the robustness of the appliance, especially as in many studies of clinical Herbst appliance robustness the time elapse until the first event (complication) was not reported, but instead an indication of fracture rates for different appliances was given. Applying the above formula, the complication rate reported by Silva et al. [16] would be reduced from 85.3 % to 44.7 %, if the reported more laborious complications (fracture or failure of crowns, splints, pivots, or trans-palatal arches, distortion, loosening, or breakage of telescope rods) were to be eliminated from the sum of all events.

Table 3 provides a summary of calculated rates of severe complications derived from different recent publications regarding Herbst appliance failure. For the WIN appliance, there was a mean complication-free treatment interval of 27.8 months that was not only on a par, but increased compared to equivalent vestibular competitors in terms of clinical sturdiness. However, one must bear in mind that the different appliances we have compared here for the purpose of discussing their robustness are equivalent in terms of their principle of action, but differ in terms of construction and components used; i.e., in some instances a comparison was made of appliances supported by mandibular splints and maxillary bands, while others were completely supported by bands or archwires. However, the comparison is considered to be legitimate as the various fixed functional appliances constitute different treatment options for the same type of malocclusion [12–14, 16, 26].

**Table 3:** Calculation of the mean interval without severe complications that would require a separate appointment for repair: The number of study subjects was multiplied by average treatment duration and the resulting cumulative treatment time was divided by the number of actual severe complications. Severe complications equal, in the case of the WIN Herbst appliance investigated, the number of re-attachments of loose attachment shells, as there were no other severe complications. Equivalently, severe complications in comparison studies included e.g., crown/splint/trans-palatal arch-failure or –fractures, or telescope rod distortions. It should be borne in mind that the appliances compared here (and also compared in some of those studies) serve the same purpose, but are markedly different in design of their components

	Vestibular appliance + Herbst				Lingual appliance + Herbst	
Appliance specifications	Upper/lower cast-splint	Upper/lower cast-splint	(A) Upper steel crowns (1^st^ molars), lower removable acrylic splint (B) Upper/Lower steel crowns (1^st^ molars)	(A) Upper steel crowns (1^st^ molars), lower removable acrylic splint (B) Upper/Lower steel crowns (1^st^ molars)	Upper/lower fully customised Incognito Herbst/bracket system	Upper/lower fully customised WIN Herbst/bracket system
Mode of appliance activation	Not reported in detail	Not reported in detail	Not reported in detail	Bite-jumping	Step-wise	Step-wise
Number of patients (n)	28	316	42	159	57	35
Frequency of severe complications (n)	94	755	77	219	159	8
Proportion of subjects with severe complications (%)	82.1	63.3	76.2	44.7	78.9	17.1
Duration of Herbst treatment (months)	6-7	7	12	12	12	6
Cumulative treatment time (months)	189	2.212	504	1.908	684	222
Mean interval without severe complications (months)	2.0	2.9	7.6	8.7	4.3	27.8
Reference	Hägg et al. 2002 [12]	Sanden et al. 2004 [14]	Moro et al. 2011 [13]	Silva et al. 2014 [16]	O’Keefe 2013 [21]	This study

Compared to a different combination of a Herbst appliance with a lingual appliance (Incognito), the fracture rate could be significantly lowered [21]. During the observation period, no loosening of screws was noted (Table 2), which may be attributed to an increased lateral degree of freedom of the appliance-guided mandible movement (Fig. 1). It also suggests itself that the separate attachment of the Herbst telescopes to the teeth, without using parts of the multi-bracket appliance such as archwires as a support, reduces the numbers of sites most likely to be affected by fractures or failure. This produces the practical clinical advantage that the WIN Herbst appliance treatment does not potentially impede multi-bracket treatment performed simultaneously. Successful treatment of non-compliance Angle Class II correction can be more reliably planned than with a combination of a band-supported Herbst appliance with a lingual appliance (Incognito). Consequently, there is no rationale for splitting treatment into two separate parts of Herbst and orthodontic, multi-bracket treatment.

### Study limitations

This study reports the proportions of complications experienced with the lingual WIN Herbst appliance, and compares the findings to proportions of complications resulting from the use of vestibular Herbst appliances. The type of appliance activation (stepwise advancement, or bite jumping) may be seen as a factor that has a potential impact on appliance fractures. However, this has not been given consideration in our comparisons as the reporting of the mode of activation has not been provided by all author groups publishing on the subject. The classical description of Herbst activation includes an initial edge-to-edge or ‚bite-jump’ activation [6, 7, 10]. This type of activation was, for instance, adopted by Silva et al. [16]. As previously reported elsewhere [14], a step-wise mode of activation (as has been performed in the patients of this trial) may potentially have an impact on the duration of getting accustomed to the appliance, as well as on lower muscular counter-force activities, and thereby may contribute to a decrease in proportions of appliance failure.

An additional limitation to generalisability of our findings may be seen in the fact that all participants of the present study have been treated in one orthodontic practice specialised in lingual orthodontic therapy, with a resulting superior qualification of the technical staff and clinicians, compared to centers that are not providing lingual orthodontic therapy, on a routine basis. On the other hand, the findings on proportions of vestibular Herbst appliance fractures have also been derived from orthodontic offices whose staffs have been trained in the use and maintainance of vestibular Herbst appliances. In general, attempts to interprete or generalise findings on proportions of orthodontic appliance failures should consider the fact the data are mostly based on treatments performed by experienced orthodontic professionals, and may differ in quality from treatment results of inexperienced teams.

## Conclusions

- During a treatment period of 10 months with the lingual Herbst appliance WIN, more than half of subjects (56.8 %) can be expected to experience no complications at all.
- Evaluated in terms of severe or treatment-intensive complications, a mean complication-free interval of 27.8 months has been calculated. Taking into consideration the step-wise mode of activation used here as well as the differences in the design of the various Herbst appliances, the Herbst appliance which we investigated appears superior to conventional band- or splint-supported appliances when compared in terms of clinical sturdiness.
- Success in treatment of non-compliant correction of Angle Class II malocclusions is considered to have better predictability using the modified anchorage strategy of the WIN Herbst appliance.
